# Sphingosine is able to prevent and eliminate *Staphylococcus epidermidis* biofilm formation on different orthopedic implant materials in vitro

**DOI:** 10.1007/s00109-019-01858-x

**Published:** 2019-12-20

**Authors:** Sascha Beck, Carolin Sehl, Sylvia Voortmann, Hedda Luise Verhasselt, Michael J. Edwards, Jan Buer, Mike Hasenberg, Erich Gulbins, Katrin Anne Becker

**Affiliations:** 1 Department of Molecular Biology, Medical School Essen, University Hospital Essen, University of Duisburg-Essen, Hufelandstrasse 55, 45122 Essen, Germany; 2 Department of Orthopedics and Orthopedic Surgery, Saarland University Medical Center and Saarland University Faculty of Medicine, Homburg, Germany; 3 Institute for Experimental Immunology and Imaging, Medical Research Center, University Hospital Essen, University of Duisburg-Essen, Essen, Germany; 4 Institute of Medical Microbiology, Medical School Essen, University Hospital Essen, University of Duisburg-Essen, Essen, Germany; 5 Department of Surgery, University of Cincinnati, Cincinnati, USA

**Keywords:** Sphingosine, Periprosthetic infection, Antibacterial effect, Biofilm

## Abstract

**Abstract:**

Periprosthetic infection (PPI) is a devastating complication in joint replacement surgery. On the background of an aging population, the number of joint replacements and associated complications is expected to increase. The capability for biofilm formation and the increasing resistance of different microbes to antibiotics have complicated the treatment of PPI, requiring the need for the development of alternative treatment options. The bactericidal effect of the naturally occurring amino alcohol sphingosine has already been reported. In our study, we demonstrate the antimicrobial efficacy of sphingosine on three different strains of biofilm producing *Staphylococcus epidermidis*, representing one of the most frequent microbes involved in PPI. In an in vitro analysis, sphingosine’s capability for prevention and treatment of biofilm-contamination on different common orthopedic implant surfaces was tested. Coating titanium implant samples with sphingosine not only prevented implant contamination but also revealed a significant reduction of biofilm formation on the implant surfaces by 99.942%. When testing the antimicrobial efficacy of sphingosine on sessile biofilm-grown *Staphylococcus epidermidis*, sphingosine solution was capable to eliminate 99.999% of the bacteria on the different implant surfaces, i.e., titanium, steel, and polymethylmethacrylate. This study provides evidence on the antimicrobial efficacy of sphingosine for both planktonic and sessile biofilm-grown *Staphylococcus epidermidis* on contaminated orthopedic implants. Sphingosine may provide an effective and cheap treatment option for prevention and reduction of infections in joint replacement surgery.

**Key messages:**

• Here we established a novel technology for prevention of implant colonization by sphingosine-coating of orthopedic implant materials.

• Sphingosine-coating of orthopedic implants prevented bacterial colonization and significantly reduced biofilm formation on implant surfaces by 99.942%.

• Moreover, sphingosine solution was capable to eliminate 99.999% of sessile biofilm-grown *Staphylococcus epidermidis* on different orthopedic implant surfaces.

## Introduction

Biofilm formation is known as the key factor for the evolution and persistence of an infection of indwelling devices [1]. Bacteria adherent to implant surfaces produce a complex-hydrated matrix of glycocalyx that coats the bacteria. This layer is called biofilm and enables bacterial colonization to evade immunological defense or antibiotic treatment [2–4]. Biofilm formation contributes to the chronicity of an infection. Although less common than an infection related to catheters, infections associated with orthopedic implants are more difficult to manage [1].

Often, these infections result in periprosthetic infections (PPI) leading to implant failure and destruction of surrounding tissue, patient disability, and on occasion, death [1, 2]. PPI is one of the main reasons for revision in joint replacement (hip 15%, knee 25%) [5].

In so-called early infections lasting only for a few days, irrigation, partial removal of the implant, and debridement are valuable treatment options. But, in infections with small colony variants or infections lasting for more than 4 weeks, removal of the contaminated implant coupled with extensive tissue and bone debridement as well as prolonged antimicrobial treatment is recommended [2, 5, 6]. Radical removal of the infected tissue and bone further complicates replantation of hardware after eradication of PPI. Moreover, radical debridement for PPI compromises function of the affected limb. Currently, single and two stage surgical treatment strategies in PPI have been developed demonstrating success rates for eradication between 81.0 and 94.6 [7].

Given that antibiotic resistance is increasing, treatment of PPI poses even greater challenges for the surgeon in the future. Therefore, alternative mechanisms to treat bacterial infections essentially have to be identified, in particular approaches that prevent infection or allow eradication without extensive additional surgery.

Sphingosine has been reported to have a bactericidal effect on a variety of Gram-negative and Gram-positive bacteria, e.g., *Pseudomonas aeruginosa*, *Acinetobacter baumannii*, *Burkholderia cepacia*, and *Staphylococcus aureus* [8, 9]. The enzyme acid sphingomyelinase catalyzes the hydrolysis of sphingomyelin to ceramide. Ceramide is further metabolized to sphingosine by acid ceramidase [10]. Recent findings suggest that a lack of sphingosine plays an important role in pneumonia, particular in cystic fibrosis patients, and delivery of sphingosine may be a novel therapy for management of pulmonary infections [9–11].

In this study, we investigate the effect of sphingosine on *Staphylococcus epidermidis*, an organism that is prevalent in orthopedic infections and notorious for forming biofilms.

## Materials and methods

### Bacteria

Three different strains of *Staphylococcus epidermidis* (*S. epidermidis*) were used. Two strains were isolated from patients (the study was approved by the local ethics committee; 19-8946-BO). One of these demonstrated resistance to oxacillin (clinically isolated oxacillin-resistant *S. epidermidis*; ORSE); the other was sensitive (clinically isolated oxacillin-sensitive *S. epidermidis*; OSSE). The third strain ATCC 51625, being resistant to methicillin, was mainly used in the experiments. Microbes were grown on fresh trypticase soy agar plates, containing 5% blood (Becton Dickinson Biosciences, Heidelberg, Germany, #254053) for 16 h at 37 °C. Bacteria were then transferred into 40 mL pre-warmed trypticase soy broth (TSB; BD Biosciences, Heidelberg, Germany, # 221093), starting with an optical density of 0.220 at 550 nm. *S. epidermidis* was grown for 75 min at 37 °C on a horizontal shaker with 125 rpm to obtain bacteria in the early logarithmic phase.

### Planktonic bacteria

To work with planktonic bacteria, bacteria were centrifuged at 2240*g* for 10 min, washed twice and diluted in HEPES buffered saline (H/S; 20 mM HEPES, 132 mM NaCl, 5 mM KCl, 1 mM CaCl_2_, 0.7 mM MgCl_2_, 0.8 mM MgSO_4_, pH 7.4).

### Biofilm generation

Biofilms of *Staphylococcus epidermidis* were generated by a 1:100 dilution of the bacteria in the early logarithmic phase in TSB, supplemented with 1% glucose, as previously described by Stepanovic et al. [12]. A total of 1 mL of this bacterial suspension was added to each well of a 24-well plate, containing a sample of implant material as indicated. Biofilms were allowed to generate for 26 h at 37 °C, normal CO_2_ atmosphere, without shaking.

### Sphingosine treatment of planktonic bacteria

To test for the antibacterial effect of sphingosine against planktonic *S. epidermidis*, bacteria were incubated in H/S with increasing concentrations of sphingosine (C18-sphingosine, Avanti Polar Lipids, Inc., AL, USA). To this end, a sphingosine stock solution of 10 mM in 0.9% NaCl was prepared by sonication and stored at − 20 °C. Stock solution was sonicated directly before use using a bath sonicator, diluted in H/S, and sonicated again for 10 min.

Bacteria were incubated with indicated sphingosine concentrations for 1 h at 37 °C on a horizontal shaker with 125 rpm. After this time, an aliquot of the bacteria was plated on Luria broth (LB) agar plates and incubated overnight to determine bacterial survival.

### Sphingosine treatment of *S. epidermidis* in biofilms

Biofilms of *S. epidermidis* were cultured as indicated above on different implant materials and incubated with increasing sphingosine concentrations in H/S for 1 h at 37 °C on a horizontal shaker. After 1 h of incubation, implant material was transferred to 1 mL of H/S, and the material was sonicated for 15 min in a bath sonicator to remove adherent biofilm bacteria. Tubes were vortexed for 5 s, released bacteria were diluted 1:100 in sterile H/S, and 10 μL aliquots were plated on fresh LB plate to determine survival of the bacteria after overnight incubation.

### Coating of implant material

Sphingosine coating of titanium and steel Kirschner wires (K-wires) was performed by a dip-coating method. Sphingosine (30 mM) was dissolved in absolute, bio-grade ethanol (Riedel-de-Haen, Seelze, Germany). The solution was agitated and tip-sonicated until no aggregates were visible anymore.

Implant materials were coated by dipping them into 70 °C pre-heated sphingosine solution for 5 s. The material was air-dried for 20 min and another consecutive dip-coating was performed.

### Ex vivo testing of coating stability

To test for mechanical coating stability, sphingosine-coated titanium K-wires were implanted into ex vivo murine femur bones. After K-wire extraction, the amount of sphingosine adherent to the titanium K-wire was analyzed with the aid of biochemical sphingosine kinase assay.

### Quantification of sphingosine-coating by biochemical sphingosine kinase assay

To determine total sphingosine amounts on sphingosine-coated implants, an enzyme kinase assay was performed. Lipids were removed from implant material by adding 200 μL H_2_0 and 800 μL extraction mix consisting of CHCl_3_:CH_3_OH:1N HCl (100:100:1; v/v/v) to each sample, followed by vortexing and centrifugation. The lower phase was collected, dried, and dissolved in 20 μL detergent solution (7.5% N-octyl-β-glucopyranoside, 5 mM cardiolipin, 1 mM DETAPAC) and sonicated for 10 min in a bath sonicator. Assay buffer (50 mM HEPES, pH 7.4; 250 mM NaCl; 30 mM MgCl_2_), 1 mM ATP, 10 μCi [^32^P]γ-ATP, and 1 mU recombinant murine sphingosine kinase (SKI; R&D) were added to start the kinase reaction. After 2 h of incubation at 37 °C and with 250 rpm shaking, the reaction was terminated by adding 20 μL 1 N HCl, 800 μL of CHCl_3_:CH_3_OH:1N HCl (100:200:1, v/v/v), 240 μL CHCl_3_, and 240 μL 2 M KCl, successively. The lower phase was collected, dried, and dissolved in 20 μL CHCl_3_:CH_3_OH (1:1, v/v). Lipids were separated by thin layer chromatography (TLC) on silica G60 plates using CHCl_3_:CH_3_OH:CH_3_COOH:H_2_0 (18:18:3:1, v/v/v/v) as developing solvent. TLC plates were exposed to radiography plates, scanned by a Fujifilm Image Analyzer (FLA-3000), and the amounts of sphingosine in the samples were calculated using a standard curve of C18 sphingosine.

### Dip infection

To mimic bacterial implant contamination, implant material samples were dipped into a solution of pre-warmed TSB with 2000 bacteria per mL. Dipping of the implant material samples was performed for 5 s, and drying of the bacterial fluid on the implant samples was waited for. The contaminated samples were then incubated in 1 mL of TSB with 1% glucose in a 24-well plate, and biofilms were allowed to generate as described above.

### Scanning electron microscopy

Biofilms of *S. epidermidis* were generated on titanium K-wires as described above. After 26 h, bacteria were fixed with 4% formaldehyde and 2.5% glutaraldehyde (in 0.1 M PHEM buffer; 60 mM PIPES, 25 mM HEPES, 10 mM EGTA, and 2 mM MgCl_2_) for 2 h at room temperature. After washing with PHEM buffer for 15 min, samples were dehydrated first in an ascending ethanol row (30%/50%/70%/80%/96%/3 × 100%; each step 15 min) followed by critical-point drying (CPD7501; Polaron/Quorum Technologies, East Sussex, UK). Subsequently, the K-wires were glued on a standard 1.9 cm aluminum SEM specimen stub using double-faced adhesive carbon tape. Ultimately, samples were coated with a 7 nm platinum/palladium layer (208HR; Cressington, Watford, UK) before SEM was conducted. Images were acquired on a dual beam SEM (Crossbeam 540; Zeiss, Oberkochen, Germany) at 2 kV acceleration voltage and 130 pA using the SE2 detector and the “High Resolution” mode of the “SmartSEM” operation software (Ver. 6.40).

### Statistics

Data are means ± SD. Statistical significance was evaluated using the GraphPad Prism 8 program performing ANOVA with Šidák as post-hoc test. *p* values less than 0.05 were considered statistically significant; details are defined in each figure legend. Graphics were created with Prism 8 and Adobe Illustrator 2019.

## Results

### Antimicrobial efficacy of sphingosine on planktonic *S. epidermidis*

**Fig. 1:**
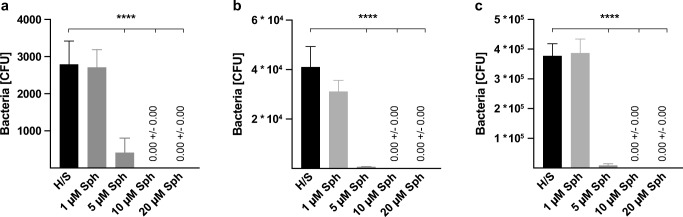
Antimicrobial efficacy of sphingosine on planktonic *S. epidermidis*. 5 × 10^3^ (**a**), 1 × 10^5^ (**b**), and 1 × 10^6^ (**c**) *S. epidermidis* (strain ATCC 51625) were incubated with indicated sphingosine (Sph) concentrations in 500 μL H/S for 1 h at 37 °C and 125 rpm. After incubation, aliquots were plated on agar plates, and the next day, colony forming units (CFU) were counted. Depicted are the total bacteria surviving the incubation in H/S or different sphingosine concentrations. Shown are mean ± SD; significant differences were compared with the H/S control using ANOVA and Šidák as post-hoc test, ****p < 0.0001

Since sphingosine was shown to have a direct anti-bacterial effect against several Gram-negative and Gram-positive bacteria in vitro [9, 13], we first tested the bactericidal efficacy of sphingosine on planktonic *S. epidermidis* ATCC 51625 bacteria. Therefore, we incubated 5 × 10^3^, 1 × 10^5^, and 1 × 10^6^*S. epidermidis* ATCC 51625 with increasing sphingosine amounts for 1 h in H/S. After incubation, 1/10, 1/100, or 1/1000 of the suspensions was plated on agar plates, respectively, and bacterial survival was determined after overnight incubation at 37 °C. Incubation of bacteria with H/S alone had no significant effect on the survival of *S. epidermidis*. Incubating *S. epidermidis* with 5 μM or higher concentrations of sphingosine revealed a significant bactericidal effect of sphingosine on 5 × 10^3^, 1 × 10^5^, and 1 × 10^6^*S. epidermidis*. The results showed that 10 μM sphingosine, equal to a total of 5 nmol of sphingosine, was able to eliminate all bacteria (Fig. 1).

### Sphingosine eliminates biofilm-grown *S. epidermidis* on different implant surfaces

**Fig. 2:**
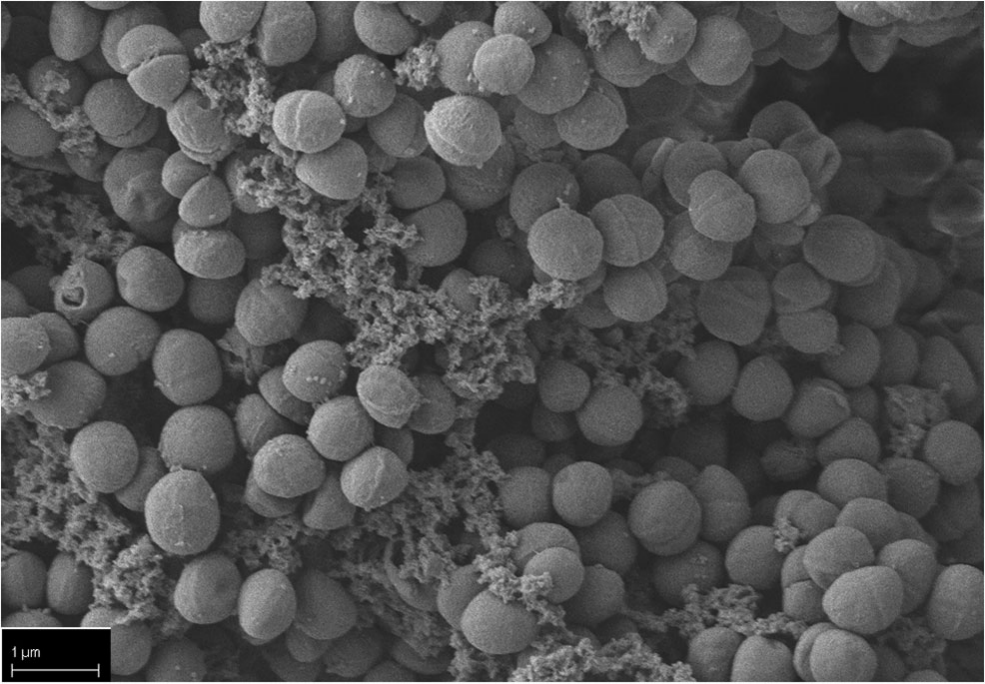
*S. epidermidis* biofilm grown on a titanium wire. *S. epidermidis* was allowed to form a biofilm on titanium wires by incubating the wires in TSB, containing 1% glucose for 26 h. Scanning electron microscopy at a magnification of 10,000 clearly visualizes coccoid cells (*S. epidermidis*) covered by an extracellular polymer matrix (biofilm). Shown is a representative example from 4 studies

**Fig. 3:**
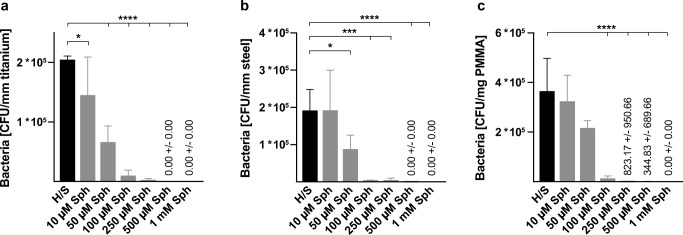
Antimicrobial efficacy of sphingosine immersion on different biofilm contaminated implant materials. Implant samples contaminated with *S. epidermidis* were immersed in increasing concentrations of sphingosine (Sph) for 1 h at 37 °C and 125 rpm. Sonication was applied to detach adherent bacteria. CFUs were determined and related to the implant size; **a** titanium, **b** steel, and **c** PMMA. Shown are mean ± SD; significant differences were compared with the respective control using ANOVA and Šidák post-hoc test; *p < 0.05, ***p < 0.001, ****p < 0.0001

In the next step, we checked for the efficacy of sphingosine on *S. epidermidis* ATCC 51625, grown in biofilms. *S. epidermidis* ATCC 51625 biofilms were generated on different implant material samples according to the method described by Stepanovic et al. [12]. As control, biofilm formation was confirmed on titanium wires using scanning electron microscopy (Fig. 2). Next, *S. epidermidis* biofilms were established on steel and titanium K-wires (diameter 1 mm) and uniform-appearing small PMMA pieces that were cut off from a clot. This was achieved by incubation of the implant samples for 26 h at 37 °C in 24-well plates with *S. epidermidis*. To test whether sphingosine kills *S. epidermidis* in these biofilms, the samples were then incubated in sphingosine or control buffer for 60 min. Finally, the implant samples were transferred to 1 mL H/S and sonicated to remove adherent biofilm-grown bacteria. Released bacteria were diluted serially in sterile H/S, and aliquots were plated on fresh LB plates to detect surviving bacteria. Bacterial counts (colony forming units, CFU) were related to the length (steel and titan wires) or weight (PMMA) of the implant samples, respectively. No difference of bacterial adhesion on titanium and steel implant surfaces was detectable (*p* = 0.632) (Fig. 3 a and b), whereas on PMMA, a significantly higher bacterial load was present (*p* = 0.003) (Fig. 3c). Sphingosine dose dependently decreased survival rates of biofilm-grown bacteria, while incubation of the contaminated implant samples in H/S did not have any significant effect on biofilm-grown *S. epidermidis* (steel, *p* = 0.105; titanium *p* = 0.603; PMMA *p* = 0.999). The antimicrobial efficacy of sphingosine on biofilm-grown *S. epidermidis* colonizing the implant samples was highly significant (*p* = 0.001) with sphingosine concentrations of 100 μM tantamount to elimination rates of at least 94% (Fig. 3). Although we selected only uniform appearing PMMA pieces, there was a higher variation in CFU counts on PMMA samples that might be due to the inhomogeneous porous surface of PMMA.

**Fig. 4:**
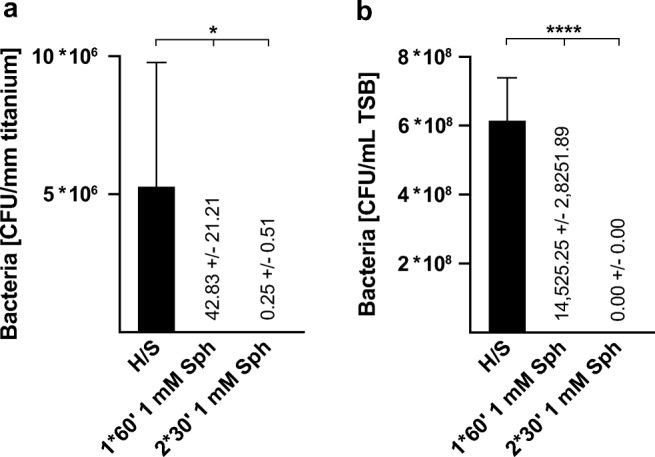
Elimination rates of sessile biofilm-grown *S. epidermidis* on titanium surfaces by immersion in 1 mM sphingosine. Biofilm-contaminated implant samples were immersed in 1 mM sphingosine (Sph) for 1 h (1*60′) or 2 times each 30 min (2*30′). Sphingosine-treated implant samples were cultured in TSB overnight at 37 °C to allow for proliferation of surviving bacteria. Both implant samples (**a**) and TSB solution (**b**) were analyzed for surviving bacteria. Shown are mean ± SD; significant differences were compared with the respective control using ANOVA and Šidák as post-hoc test; *p < 0.05, ****p < 0.0001

Immersing the biofilm-containing steel, titanium or PMMA implants in 1 mM sphingosine suspension for 1 h resulted in bacterial counts that were below detection levels. To absolutely exclude any bacterial growth, we treated biofilm-containing titanium samples with 1 mM sphingosine and then allowed any remaining bacteria to re-grow by incubation of the titanium samples in TSB overnight. The TSB solution and the implant samples were then analyzed for bacterial survival. Thus, even a single remaining bacteria would be detected, since the generation time of *S. epidermidis* in TSB has been reported to be between 40 and 50 min [14]. The results revealed that 1 mM sphingosine treatment eliminated 99.999% of the biofilm-grown *S. epidermidis* on titanium implant surface, compared with controls. Sphingosine was even more effective, when replacing the sphingosine solution once after 30 min (Fig. 4a).

Moreover, TSB solution was completely sterile after immersing the implants twice for 30 min in 1 mM sphingosine. Therefore, sphingosine immersion not only prevented biofilm-growth of *S. epidermidis* and in fact killed the pathogen in the biofilm, but also prevented transition of adherent bacteria into its planktonic condition (Fig. 4b).

**Fig. 5:**
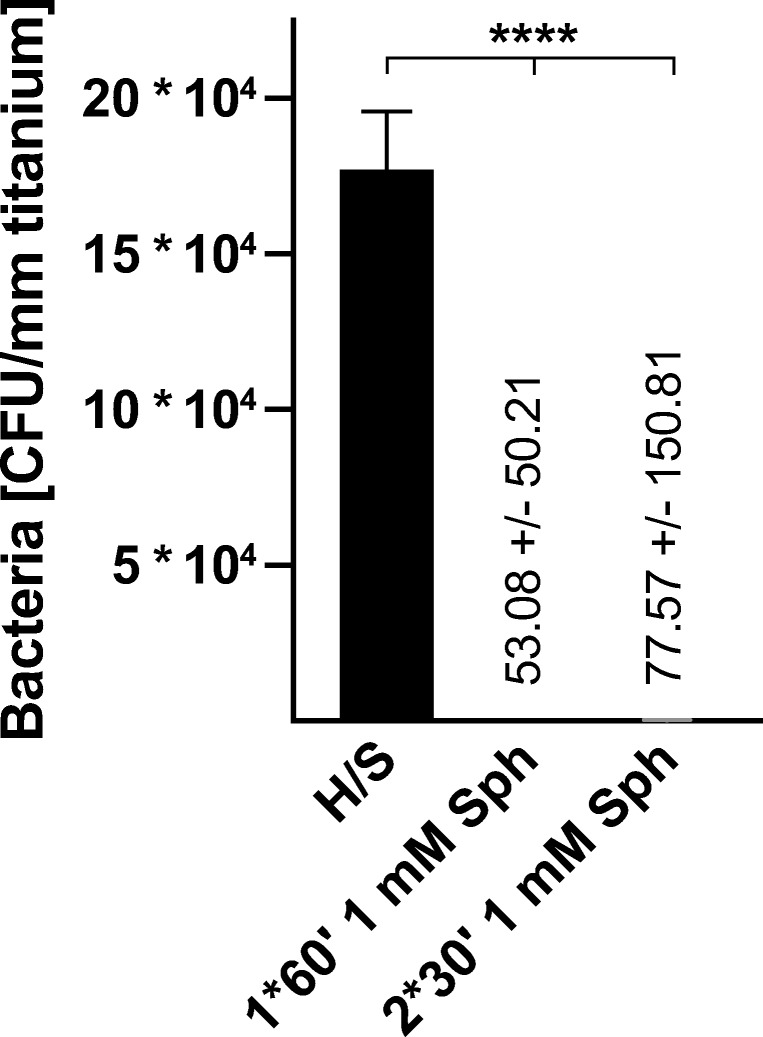
Elimination rates of sessile biofilm-grown *S. epidermidis* without sonication. To exclude any influence of sonication on bacterial survival, *S. epidermidis* (strain ATCC 51625) biofilm-contaminated titanium implant samples were immersed in 1 mM sphingosine for 1 h. After sphingosine treatment, implant samples were transferred into TSB for 4 h at 37 °C and 125 rpm to allow re-growth of biofilm bacteria and transition into planktonic condition. TSB was analyzed for planktonic *S. epidermidis*. Shown are mean ± SD; significant differences were compared with the respective control using ANOVA and Šidák as post-hoc test; ****p < 0.0001

To exclude any interference of the sonication process on bacteria, biofilm-contaminated titanium samples were immersed in 1 mM sphingosine for 1 h as described above. After sphingosine treatment, the implant samples were incubated in TSB again at 37 °C and 125 rpm to allow re-growth of biofilm bacteria and transition into the planktonic condition. After 4 h of incubation, TSB was analyzed for planktonic bacteria. TSB with sphingosine-treated samples revealed only 0.0003% of bacterial counts compared with TSB with untreated samples. These results demonstrate that sonication has no additional negative effect on bacterial survival in the experimental approach (Fig. 5).

**Fig. 6:**
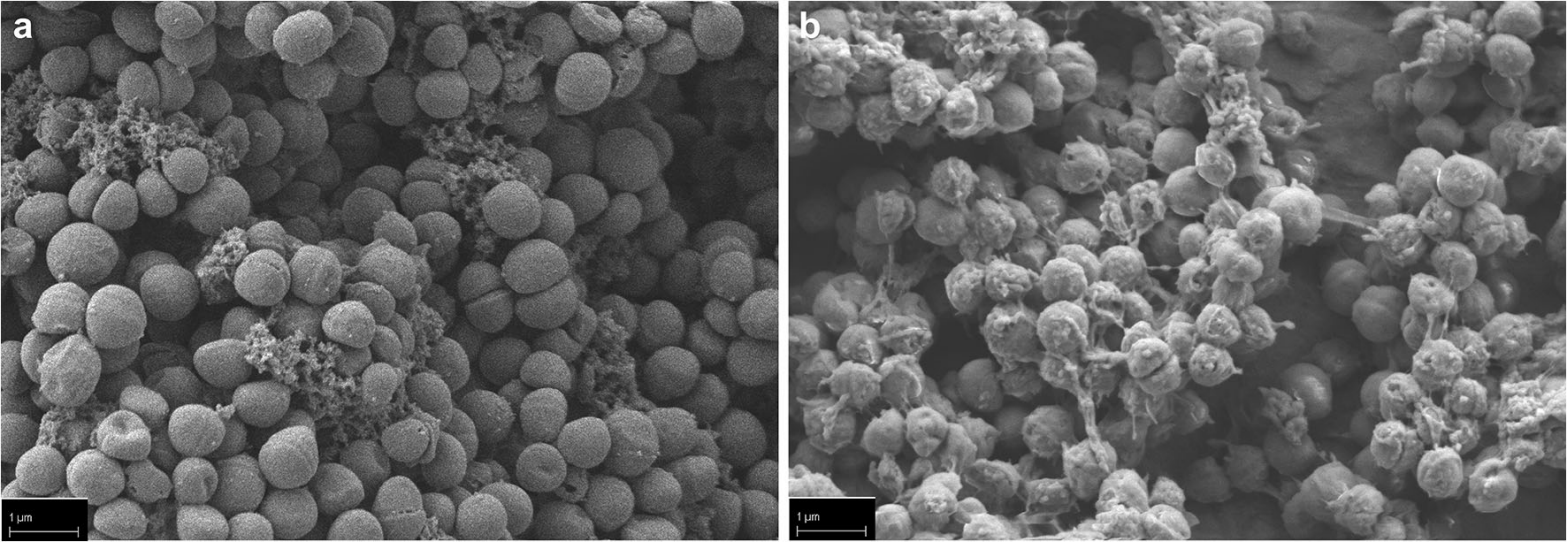
Sphingosine treatment induces ultrastructural damage of biofilm grown *S. epidermidis* on a titanium wire. *S. epidermidis* (strain ATCC 51625) was allowed to form a biofilm on titanium wires by incubating the wires in TSB, containing 1% glucose for 26 h. Scanning electron microscopy at a magnification of 10,000 clearly visualizes (**a**) coccoid cells covered by an extracellular polymer matrix (biofilm) and (**b**) *S. epidermidis* biofilm after treatment with 1 mM sphingosine. After sphingosine treatment, the coccoid cells appear distorted with concave and crumpled surfaces. Shown is a representative example from 4 studies.

Our data indicate that sphingosine is highly effective for eradication of biofilm-grown bacteria. Scanning electron microscopy (SEM) of biofilm-contaminated titanium samples after sphingosine treatment still revealed a bacterial layer covering the sample. But, in contrast to the untreated samples (Fig. 6a), sphingosine induced distorted and crumpled surfaces of the *S. epidermidis* ATCC 51625 coccoid cells (Fig. 6b) indicating ultrastructural damage to the adherent microbes.

### Sphingosine reduces *S. epidermidis* biofilm formation on titanium implant surface

**Fig. 7:**
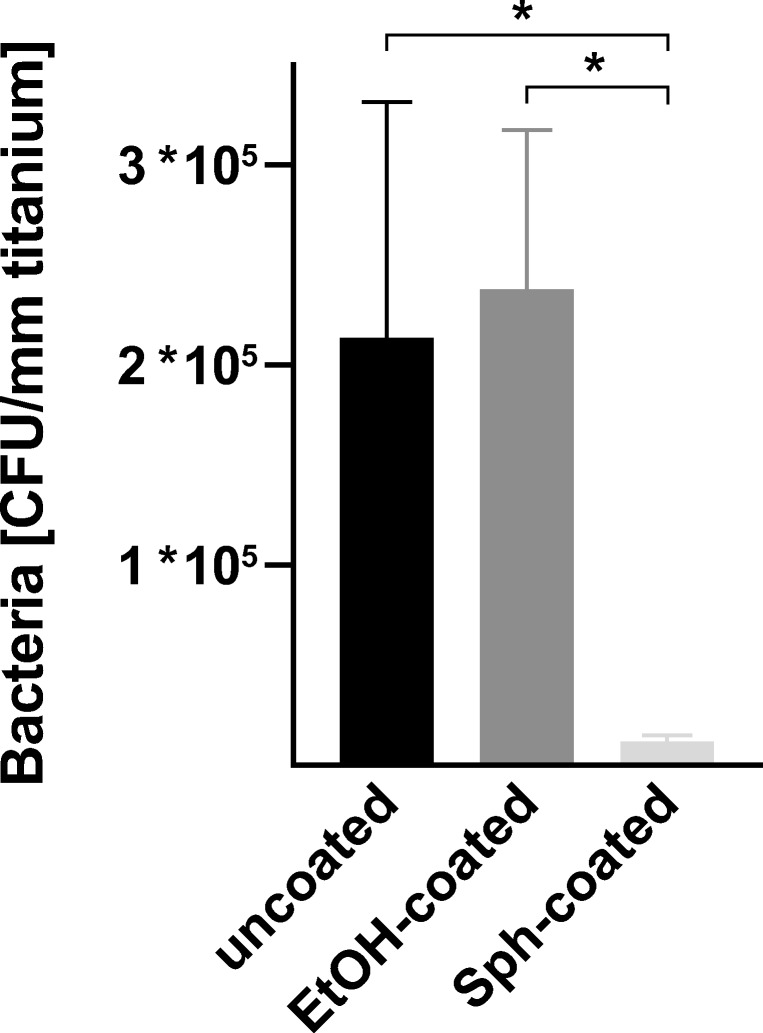
Sphingosine-coating reduces biofilm formation on titanium implant surface. Sphingosine (Sph)- or control-ethanol–coated titanium implant samples were cultured in TSB, containing 1% glucose for 26 h at 37 °C to allow biofilm formation. The next day, sonication was applied to detach adherent bacteria. CFUs were determined and related to the size of the titanium samples. Shown are mean ± SD; significant differences were compared with the respective control using ANOVA and Šidák as post-hoc test; *p < 0.05

Having examined the antimicrobial efficacy on biofilm-grown *S. epidermidis* ATCC 51625, we examined sphingosine’s capability to prevent implant contamination by coating the implant surfaces. To this end, titanium implant samples were dip-coated twice in sphingosine or, as control ethanol, to ensure complete implant coating. The coated implant samples were then cultured for biofilm formation as described above. After 26 h, the titanium implant samples were transferred into an Eppendorf reaction tube, containing 1 mL of H/S, and were sonicated for 15 min to remove adherent biofilm-grown bacteria. Released bacteria were serially diluted in sterile H/S, and aliquots were plated on agar plates to determine the amount of biofilm formation. CFUs were related to the length of the implant samples. Figure 7 demonstrates the effect of sphingosine-coating on bacterial biofilm formation and growth. Sphingosine-coating of the titanium resulted in a reduction of biofilm formation on the implant by 99.942%, while coating titanium with ethanol was without effect on biofilm formation (*p* = 0.774).

### Sphingosine-coating prevents titanium implant contamination

**Fig. 8:**
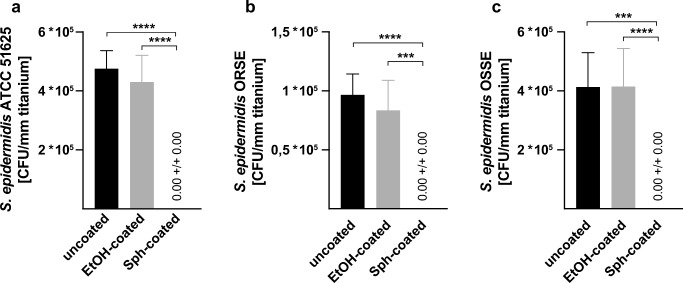
Sphingosine-coating prevents implant contamination of titanium samples. To mimic bacterial implant contamination, sphingosine-coated titanium implant samples were dip-infected in a solution containing 2000 bacteria per mL of three different *S. epidermidis* strains: ATCC 51625 (**a**), ORSE (**b**), and OSSE (**c**). Contaminated implants were transferred to TSB/1% glucose for 26 h to allow biofilm formation. Implant contamination (CFU) was determined and related to the size of the titanium implant. Shown are mean ± SD; significant differences were compared with the respective control using ANOVA and Šidák as post-hoc test; ***p < 0.001, ****p < 0.0001

**Fig. 9:**
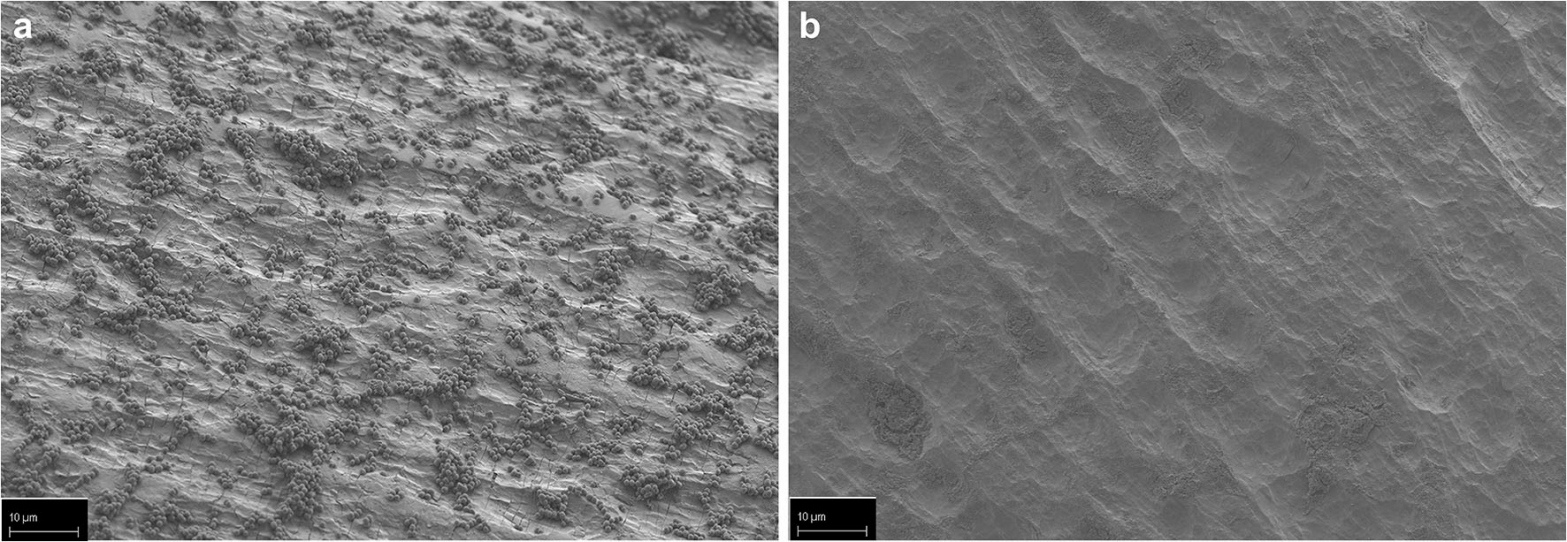
Prevention of implant contamination by sphingosine-coating visualized in SEM. Uncoated and sphingosine-coated titanium implant samples were dip infected with *S. epidermidis* (strain ATCC 51625) and incubated 26 h in TSB containing 1% glucose to allow biofilm formation. SEM at a magnification of 1000 clearly visualizes scattered *S. epidermidis* colonies covering the uncoated wire (**a**), whereas no bacterial colonization could be detected on the sphingosine coated implant (**b**)

Next, we tested the efficacy of sphingosine to prevent implant contamination on another infection route: titanium implants were coated with sphingosine as described above. Implant contamination was mimicked by dipping the uncoated, control- or sphingosine-coated titanium samples into a solution of TSB with 2000 bacteria of different *S. epidermidis* strains (ATCC 51625, ORSE, and OSSE) per mL for 5 s (Fig. 8a–c). Contaminated implants were immersed in TSB with 1% glucose to allow biofilm formation on the titanium surface. After 26 h, the implant samples were transferred into 1 mL H/S and sonicated to release biofilm-grown bacteria, diluted in H/S, and aliquots were plated. CFUs were related to implant length. Sphingosine-coating entirely prevented implant contamination, irrespective of the *S. epidermidis* strain (Fig. 8). The antimicrobial efficacy of sphingosine-coating could also be demonstrated by SEM analysis (Fig. 9).

### Stability of sphingosine-coating

**Fig. 10:**
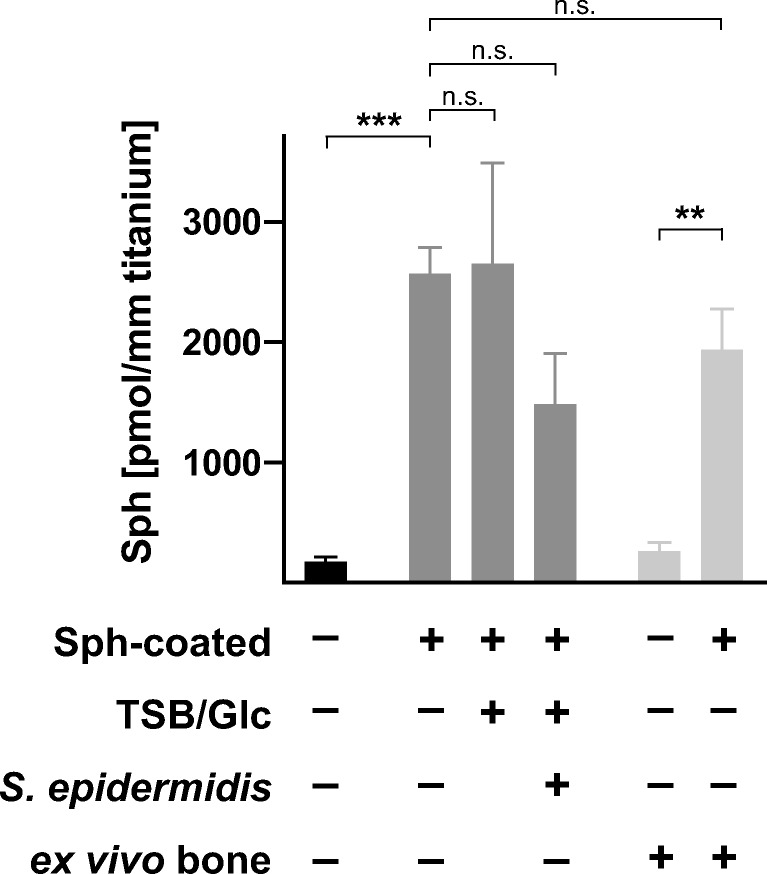
Stability of sphingosine-coating. Sphingosine-coating stability in fluids was verified. Sphingosine-coated titanium implant samples were immersed in TSB, containing 1% glucose (TSB/Glc) or TSB/Glc, containing *S. epidermidis* ATCC 51625 (*S.e.*), for 26 h. Mechanical stability of sphingosine (Sph) was confirmed by implanting sphingosine-coated titanium K-wires ex vivo into murine femur bones. Shown are mean ± SD; significant differences were compared with the respective control using ANOVA and Šidák as post-hoc test; **p < 0.01, ***p < 0. 001

To analyze the efficacy and stability of sphingosine-coating, titanium K-wires were dip-coated twice in sphingosine, and sphingosine was quantified by a kinase assay. These analyses revealed that the implants were coated with an amount of 2.567 ± 0.223 nmol sphingosine/mm titanium K-wire implant (0.6 mm diameter). The stability of sphingosine-coating in fluids was assessed by immersing coated titanium implant samples for 26 h in solutions applied for biofilm generation (TSB + 1% glucose with or without *S. epidermidis* ATCC 51625). Sphingosine-coating proved to be stable in fluids with no significant reduction of sphingosine on the implant surface for at least 26 h of incubation. Incubation in TSB solution containing *S. epidermidis* ATCC 51625 revealed slightly reduced amounts of sphingosine on the implant samples. Although the effect was not significant, these results suggest that sphingosine is consumed when releasing its bactericidal effect. To verify mechanical stability, sphingosine-coated titanium K-wires were implanted into ex vivo murine femur bones, explanted again, and the amount of sphingosine adherent to the surface was analyzed. Compared with the respective controls, implanting did not result in a significant change of sphingosine on the K-wire surface, indicating sufficient mechanical stability for the use of sphingosine as an antimicrobial coating of orthopedic implants (Fig. 10).

## Discussion

In joint replacement, poor osseointegration and infection are the main reasons for implant failure and revision surgery. Nowadays, implant infection has been reported to be the new leading cause of orthopedic implant removal, overtaking aseptic loosening [15–18].

Despite the biocompatibility of titanium and its alloys used for implants, bacteria easily colonize these surfaces. After adherence on the titanium surface, bacteria begin to proliferate, eventually reaching a high enough density to form a biofilm [18].

Often, these implant infections result in PPI, a devastating complication leading to implant failure and destruction of surrounding tissue, patient disability, and occasionally death [1, 2]. In an aging population, the amount of joint replacements and associated complications like PPI will even increase. In addition to the likely increase in incidence, treatment of PPI will even become more challenging in the face of increasing antibiotic resistance of the microbes.

*S. epidermidis* represents one of the most frequent microbes involved in PPI [19–21]. The capability for biofilm formation and the increasing resistance of *S. epidermidis* to antibiotics have complicated the treatment of colonized implants and tissues [5, 19]. Therefore, the development and implementation of more effective prevention and treatment strategies are mandatory.

From a clinical perspective, infections occurring within the first 6 weeks are typically caused by implant contamination at the time of surgery. The first 6 h after surgery are considered the most vulnerable time interval for the onset of an infection [22]. Coating the implant surfaces with bactericidal agents that can actively kill bacteria as they contact the implant surfaces is one possibility to defend early infection [18]. Bactericidal coatings may help to eliminate the introduced microbes before rapid proliferation begins. Implant coating with sphingosine is in line with this concept.

In our study, we mimicked perioperative implant contamination with three different *S. epidermidis* strains in the dip-infection experiment. Sphingosine-coating completely prevented bacterial implant contamination irrespective of the *S. epidermidis* strain. Even though, bacterial load in dip-infection was only 2000 bacteria/mL; sphingosine-coating also proved its efficacy at higher bacterial concentrations. When incubated in a solution of TSB, 1% glucose and approximately 30 million bacteria at the starting point for biofilm generation, sphingosine-coating was capable to reduce implant contamination by 99.942%.

Coating the titanium K-wires with sphingosine resulted in an average amount of 2.6 nmol sphingosine/mm implant, that remained attached for at least 26 h in fluid. When investigating the antimicrobial capacity of sphingosine solution on planktonic *S. epidermidis* ATCC 51625, we observed that an amount of 5 nmol sphingosine was sufficient for eradication of 1 × 10^6^ bacteria. Remarkably, the amount of sphingosine adherent to the implant surface by coating was capable to prevent implant contamination even when immersed in *S. epidermidis* ATCC 51625 solutions containing a bacterial load of 30 million microbes. Based on our results, we speculate that only a small amount of the sphingosine-coating was consumed upon incubation in bacterial solution. The majority of the coating remained attached on the implant surface exerting a bactericidal effect for at least 26 h. Moreover, the sphingosine-coating demonstrated good mechanical stability upon implantation of coated implants in murine femur bones.

However, implant coating has to be evaluated not only for its stability and ability to prevent infections but also for its effect on osseointegration. In this regard, it is interesting to note that sphingosine has also been implied in bone remodeling [23–25]. Therefore, sphingosine-coating of the surface might also be beneficial in osseointegration of orthopedic implants.

In implant infections lasting for more than 4 weeks, biofilm formation is complete and a PPI has developed [5]. In these cases, removal of the contaminated implant, extensive bone, and tissue debridement as well as prolonged antimicrobial treatment is recommended, demonstrating success rates for eradication between 81.0 and 94.6% [2, 5–7]. Our data show that sphingosine not only proved to be effective in preventing bacterial implant contamination, but also demonstrated its antimicrobial efficacy on biofilm-grown *S. epidermidis*. Sphingosine solution was capable to penetrate biofilm and eliminate sessile biofilm-grown *S. epidermidis* ATCC 51625 on different implant surfaces. Immersing the implant samples with matured *S. epidermidis* biofilm in 1 mM sphingosine was sufficient to eliminate 99.999% of the adherent bacteria. Replacing the sphingosine solution after 30 min even resulted in a complete eradication of sessile biofilm-grown *S. epidermidis*. To our knowledge, there are only a few agents that are capable to penetrate biofilm and exhibit an antimicrobial efficacy as described [26, 27].

Nevertheless, sphingosine’s ideal concentration and method of application to eliminate biofilm-grown bacteria and its effect on host tissue and penetration depth in vivo have to be examined.

Similar to the findings of Fischer et al. [28], investigating the effect of sphingosine on *Escherichia coli* and *Staphylococcus aureus*, SEM of biofilm contaminated implant samples after sphingosine treatment revealed distorted *S. epidermidis* ATCC 51625 surfaces indicating ultrastructural damage and cell death. The molecular mechanism of sphingosine’s bactericidal effect has not yet been fully resolved in detail. It is known that micellar sphingosine kills many pathogens including *Escherichia coli*, *Pseudomonas aeruginosa*, *Staphylococcus aureus*, *Acinetobacter baumannii*, *Moraxella catarrhalis*, *Haemophilus influenzae*, *Burkholderia cepacia*, *Neisseria meningitides*, and *Neisseria gonorrhoeae* [9, 29–34]. It is possible that sphingosine simply kills pathogens by its biophysical properties, which would also suggest that sphingosine’s antimicrobial mechanism is not prone to the development of bacterial resistance. On the other hand, bacteria express sphingosine responsive elements [35], suggesting that sphingosine may also have some biochemical effects in bacteria.

## Conclusions

Our data demonstrate that the naturally occurring amino-alcohol sphingosine is highly efficacious in the treatment of *S. epidermidis*. Sphingosine-coating prevents adherence of *S. epidermidis* to implant surfaces by a bactericidal effect of sphingosine. Moreover, sphingosine solution is capable to eliminate sessile biofilm-grown *S. epidermidis* on different implant surfaces. Nevertheless, for determination of coating stability, effects on host tissue, and penetration depth in vivo, future animal studies are necessary to establish the safety of sphingolipid treatment, and future-randomized clinical trials will be necessary to determine sphingosine’s ability to provide a cost-effective treatment strategy to reduce infection rates and improve success rates for eradication in joint replacement surgery.

## Abbreviations

CFU: Colony forming units
H/S: HEPES buffered saline
LB: Luria broth
ORSE: Clinically isolated oxacillin-resistant *S. epidermidis* strain
OSSE: Clinically isolated oxacillin-sensitive *S. epidermidis* strain
PMMA: Polymethylmethacrylate
PPI: Periprosthetic infection
SEM: Scanning electron microscopy
*S. epidermidis*: *Staphylococcus epidermidis*
TSB: Trypticase soy broth
